# Ultrasound-targeted microbubble destruction improves the suppression and magnetic resonance imaging of pancreatic cancer with polyethyleneimine nanogels

**DOI:** 10.7150/jca.93802

**Published:** 2024-03-25

**Authors:** Yang Liu, Yuanqiong Deng, Paul E Constanthin, Fan Li

**Affiliations:** 1 Department of Ultrasound, Shanghai General Hospital, Shanghai Jiaotong University School of Medicine, Shanghai, China.; 2 Department of Ultrasound, Maternal and Child Health Hospital of Shanghai Jiading District, Shanghai, China.; 3 CHU Pellegrin, Service de Neurochirurgie B, Hôpital Pellegrin-Tripode, Place Amélie Raba-Léon, 33 076, Bordeaux Cedex, France.

**Keywords:** UTMD, polyethyleneimine nanogels, pancreatic cancer, MRI

## Abstract

The chemoresistance of pancreatic cancer tumors urgently needs to be addressed. Pancreatic cancer is characterized by an abundant stroma, with significant fibrous connective tissue formation that encapsulates the tumor parenchyma and forms an interstitial microenvironment. Pancreatic stellate cells (PSCs) play a crucial role in this microenvironment and specially secrete periosteal protein (periostin), which can promote tumor growth, metastasis, and chemoresistance. Therefore, periostin has become a specific target of chemotherapy resistance intervention methods. The proposed polyethyleneimine (PEI) nanogels have multiple modification and efficient drug-loading properties. Additionally, ultrasound-targeted microbubble destruction (UTMD) supports the breakdown of the tough interstitial barrier of pancreatic cancer. A small interfering RNA (siRNA) can be used to downregulated the periostin gene, while sustained release of gemcitabine can promote killing of tumor cells. This method achieves a combination of gene silencing and chemotherapy. The imaging effect can be evaluated using magnetic resonance imaging (MRI). The ultimate goal of this work is to support individualized and effective therapeutic methods and help develop new strategies for pancreatic cancer treatment.

## Introduction

Pancreatic ductal adenocarcinoma (PDAC) is an extremely malignant type of cancer with poor prognosis and strong resistance to the first-line chemotherapy drug gemcitabine^1^-^3^. The five-year survival rate of PDAC patients is less than 5%, with a median survival time of less than six months^4^, ^5^. The inefficient delivery of chemotherapeutic drugs in PDAC is the main reason why such drugs can be ineffective for pancreatic cancer^6^-^9^. Thus, reducing the resistance to chemotherapeutic drugs and improving their delivery efficiency in pancreatic cancer cells (PCCs) are important goals for the treatment of PDAC^10^-^13^.

PDAC is often characterized by an abundant stroma^14^, ^15^. A large amount of fibrous connective tissue forms and wraps the tumor in the stroma, which constitutes the interstitial microenvironment of pancreatic cancer^16^-^18^. The lack of blood supply hinders chemotherapeutic drug delivery^19^-^21^. Pancreatic stellate cells (PSCs) can specifically secrete periostin and play a vital role in the interstitial microenvironment^22^, ^23^. Periostin mRNA expression levels in pancreatic cancer are 42 times that of normal pancreatic tissue levels, and it is specifically expressed in the stroma^24^. We previously elucidated that periostin can promote the proliferation, migration, and invasion of PCCs, as well as support subcutaneous tumor formation and abdominal metastasis of pancreatic cancer in nude mice^23^, ^25^. Additionally, periostin can significantly protect PCCs from gemcitabine-induced DNA damage, enhance the resistance of PCCs to gemcitabine, and inhibit tumor cell apoptosis^26^.

Currently, the method most commonly used worldwide to overcome the interstitial barrier of pancreatic cancer is to degrade hyaluronic acid in the stroma, which enlarges the gap between vascular endothelial cells and thereby promotes drug delivery^27^. We used a new biomechanical technology called ultrasound targeted microbubble destruction (UTMD), which is a physical method of ultrasound "punching" technology. The ultrasound microbubble contrast agent acts as a "cavitation nucleus" to enhance the ultrasound biological effect. The microbubbles rapidly expand, contract, squeeze, and rub against each other alternately under ultrasonic irradiation, then finally rupture. As a result, shock waves form instantaneous and reversible pores on the cell membrane, which allows the vector being delivered to overcome the interstitial barrier and enter the tumor cells through the acoustic pores^28^, ^29^. UTMD is expected to provide a safe and effective technical means for the sensitization of pancreatic cancer chemotherapy^30^. Therefore, this approach could revolutionize the treatment of pancreatic cancer by breaking the tough interstitial barrier, reducing structural obstacles, and supporting efficient delivery of chemotherapeutic vectors^31^, ^32^.

RNA interference silencing is one of the main cancer therapeutic methods *in vitro* experiments33. Through the complementary combination of synthetic oligonucleotide fragments and oncogene-encoded mRNA to inhibit mRNA transcription, oncogene activity can be blocked^34^, ^35^. The key issue affecting RNA interference silencing is the choice of vector. Therefore, the development of a safe and efficient vector system is a substantial research challenge. At present, the commonly used gene carriers in experimental research mainly include viruses and liposomes^36^, ^37^. Although viral vectors have high transfection efficiency, they have poor biological safety, small size, and can only carry a limited number of genes^38^. Liposomes can carry many genes, but the liposome-gene system is unstable and the tissue specificity is poor^39^. In recent years, the rapid development of nanotechnology has led to breakthrough technological innovations for gene silencing. However, the preparation of nanomaterials with high gene transfection rates and efficient tumor cell targeting remains difficult.

Polyethyleneimine (PEI) is a natural degradable polymer that has been widely used in drug delivery and tissue engineering and can be used as an ideal nanohydrogel carrier material^40^, ^41^. Nanohydrogel is a hydrophilic or amphiphilic polymer that contains multifunctional groups. It has good flexibility and fluidity and can be taken up by tumor cells more easily^42^. The characteristics of nanohydrogel include high water content, which can be changed by external conditions to shrink or expand, as well as a three-dimensional internal network structure that can encapsulate drugs. Additionally, it has a smaller particle size with a larger specific surface area that is conducive to biological coupling, thereby prolonging the blood circulation time and passive targeting^43^. Therefore, nanohydrogel has a long blood circulation time, good biocompatibility, high load capacity, good stability, and is also biodegradable and easy to synthesize^44^.

In this study, we used UTMD to promote the PEI nanohydrogel carrier by breaking the PDAC interstitial barrier and directly acting on the tumor parenchyma. Small interfering RNA (siRNA)-mediated knockdown of periostin expression in the PDAC interstitium could thereby block activity of the downstream signaling pathway and promote PCCs apoptosis. This supported chemotherapeutic drug delivery to the tumor. Gemcitabine could directly act on PCCs, with sustained release of this drug leading to increased PCC death and a reduction of chemotherapy resistance. Finally, magnetic resonance imaging (MRI) was used to achieve molecular level imaging of pancreatic cancer. Through a combination of gene silencing and chemotherapy approaches, the goal of developing a precise and visualized treatment method for pancreatic cancer can be achieved.

## Methods and Materials

### The synthesis of PEI nanohydrogels

**Figure 1:**
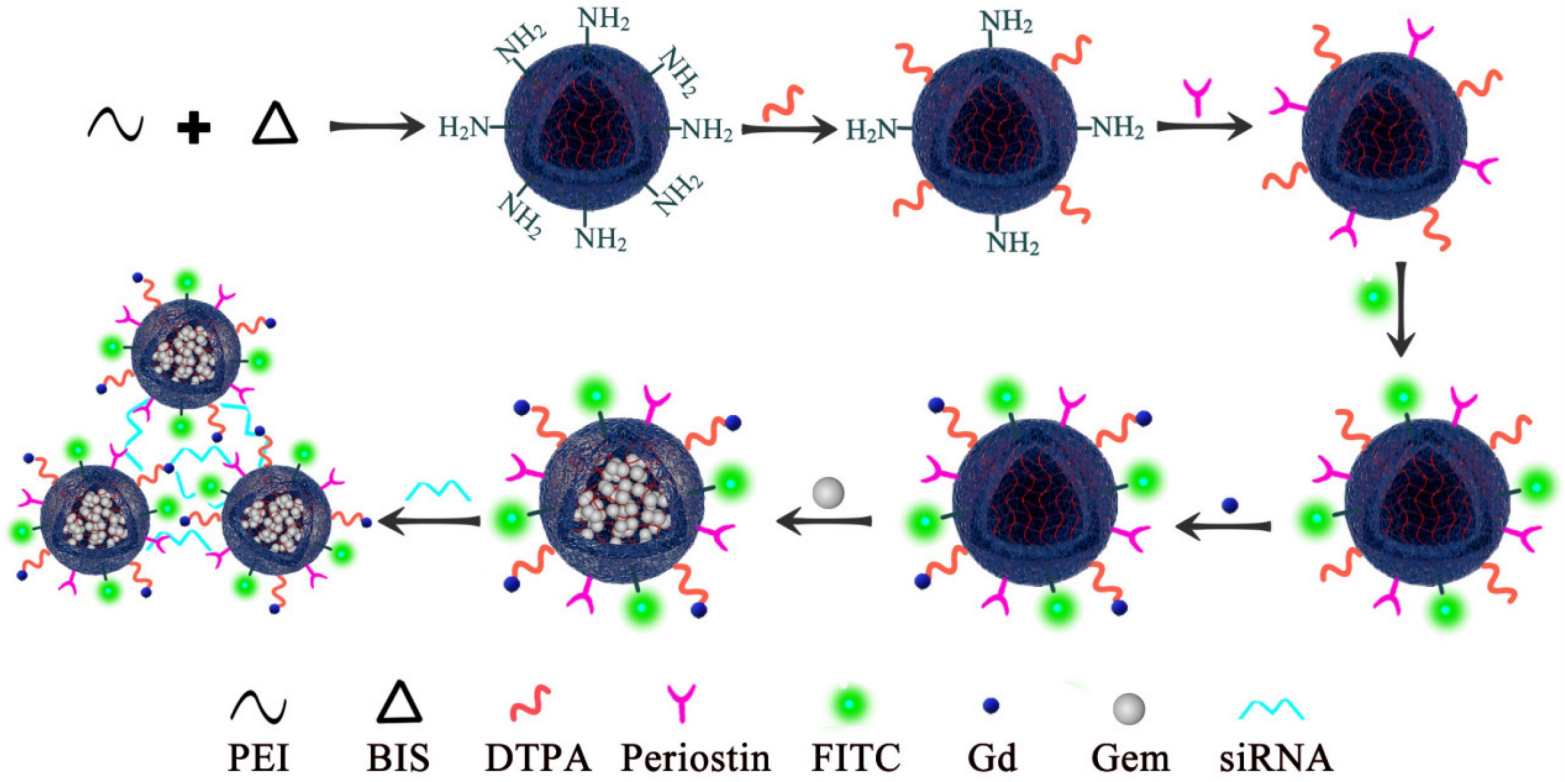
** Polyethyleneimine (PEI) nanohydrogel synthesis.** We first covalently bound the specific periostin antibody on the surface of the PEI nanohydrogel. The surface was then conjugated with fluorescein isothiocyanate (FITC). Gemcitabine was then efficiently loaded in the PEI nanohydrogel carrier, followed by the gadolinium (Gd) ion chelate being modified on the surface of the nanocarrier.

PEI nanogels were synthesized by an inverse emulsion method through a Michael addition cross-linking reaction. Briefly, branched PEI (272 mg) and N, N'-methylenebis(acrylamide) (BIS, 32 mg) were codissolved in 5 mL of water and added dropwise into a Span 80 solution (2%, w/v, in toluene) under stirring at 1000 rpm for 30 min. After that, the mixture was emulsified utilizing a Misonix Sonicator (XL2000, Division of Q Sonica, LLC., Newtown, CT) at an output wattage of 20 W for 5 min and dropwise added with 1.2 mL of triethylamine (TEA) to initiate the cross-linking reaction between BIS and PEI amines under stirring at room temperature overnight. The formed raw PEI nanohydrogels were purified by centrifugation (12,000 rpm, 15 min) and redispersed in 30 mL of methanol, followed by dialysis against water using a regenerated cellulose dialysis membrane with a molecular weight cutoff (MWCO) of 8000-14,000 for 3 days (2 L, 6 times). Upon lyophilization, the obtained PEI nanohydrogels powder was obtained and kept at -20°C before further use. Next, Diethylenetriamine penta-acetic acid (DTPA) dianhydride (40 mg) were codissolved in 6 mL of water under magnetic stirring for 12 h at room temperature and dropwise added to 15 mL of PEI nanohydrogels solution (9.4 mg/mL) under stirring at 500 rpm for 12 h. Next, we then covalently bound the specific periostin antibody on the surface of the PEI nanohydrogels. The surface was conjugated with fluorescein isothiocyanate (FITC). Gemcitabine was then efficiently loaded in the PEI nanohydrogel carrier, followed by the gadolinium (Gd) ion chelate being modified on the surface of the nanocarrier. Finally, the nanoparticles were aggregated together by the electrostatic attraction of periostin siRNA (Fig. 1).

### PEI nanohydrogel biological safety was verified using hematoxylin and eosin (H&E) staining

To study the metabolism of the PEI nanohydrogel *in vivo*, we injected PEI nanohydrogel in PBS into nude mice through the tail vein. Different time points (0, 7, 14, and 30 days) were selected for H&E staining of various tissues.

### UTMD-mediated PEI nanohydrogel carrier for imaging and treatment parameters optimization *in vivo* and *in vitro*

Many studies have shown that the fluorescent substance FITC can directly penetrate the cytoplasm through the reversible "sound pores" induced by UTMD. Therefore, this feature can be used to study the cell uptake efficiency and induced effect of UTMD. To detect the best parameters for UTMD to promote the uptake of nanoparticles by cells, we chose different ultrasonic power variable parameters (Control, 0.4W/cm^2^, and 0.6W/cm^2^) for the experiments. The FITC uptake efficiency was detected by Flow cytometry with SW1990. Quantitative analysis of FITC uptake rate and cell survival rate of SW1990 were performed after treatment with different ultrasonic powers. The FITC uptake efficiency can reflect the number of cells that took up FITC following UTMD. Finally, we aimed to maximize the cellular uptake rate and reduce cell apoptosis levels to select the best UTMD parameters.

### Cell Counting Kit-8 (CCK-8) assay

The CCK-8 cell viability assay was used to examine the effect of PEI nanohydrogel on PCCs proliferation. Four experimental groups were used: control group (mock), gemcitabine alone group (G), gemcitabine and siRNA nanohydrogel group (PEI), gemcitabine and siRNA nanohydrogel + UTMD group (PEI+UTMD). First, 3×10^3^ PCCs from each of the different treatment groups were plated in wells of a 96-well plate. After incubating for 0, 24, 48, 72, or 96 hours, 10 μL of CCK-8 solution was added to each well. The plate was then incubated at 37℃ for 2 hours. The optical density (OD) of each well was measured at 450 nm with a microplate reader. These values were used to calculate the relative proliferation rates of the cells.

### Tumor xenograft model and tumorigenicity assay

Male 4-week-old BALB/c nude mice (weight, approximately 20 grams) were obtained from Shanghai Slack Laboratory Animal Co. Ltd. SW1990 cells (3×10^6^ cells) and PSCs (3×10^6^ cells) in 20 μL of cell suspension were subcutaneously implanted into the back of nude mice. Mice were randomly divided into four groups: mock, G, PEI, PEI+UTMD (n = 5 mice/group). The tumor volume was examined weekly by a caliper and calculated using the formula: volume = 0.5×length×width^2^. After about 4 weeks, the nude mice were euthanized. These xenograft tumors were then harvested, weighed, and fixed in 10% formalin.

### Contrast Enhanced Ultrasound (CEUS) was used to observe the blood perfusion of tumors in each group

Examining the blood perfusion in the tumor after treatment is also an effective indicator for evaluating the therapeutic effect. CEUS images can show the perfusion in real time and have been used to clinically evaluate the distribution and perfusion of microvessels in tumors after treatment. To achieve this, 100 μL of SonoVue (Bracco, Italy) was injected through the tail vein while simultaneously performing CEUS to observe the blood perfusion of the tumor and quantitatively analyze it.

### The MRI effect of PEI nanohydrogels *in vivo*

The MRI effect of tumors was evaluated by intratumoral injection of PEI nanohydrogels in PBS. The experiment was divided into control group (Mock) and nanohydrogels groups (PEI and PEI+UTMD), with five nude mice in each group. All the groups were scanned with T1 sequence. We aimed to confirm that the nanohydrogel carrier combined with UTMD and MRI will help support and improve the diagnosis and treatment visualization of pancreatic tumors.

### Statistical analysis

Statistical analyses were performed using SPSS 19.0 software. Statistical comparisons were conducted by Student's t-test and the results are presented as mean±standard deviation (SD) from at least three separate experiments. A P-value of 0.05 or less was considered statistically significant.

## Results

### The entrapment efficiency and drug loading of PEI nanohydrogel

Gemcitabine (entrapment efficiency 46.01% and drug loading 21.64%) was efficiently loaded in the PEI nanohydrogel carrier with slow release of the drug maintained to continuously kill PCCs.

### H&E staining

**Figure 2:**
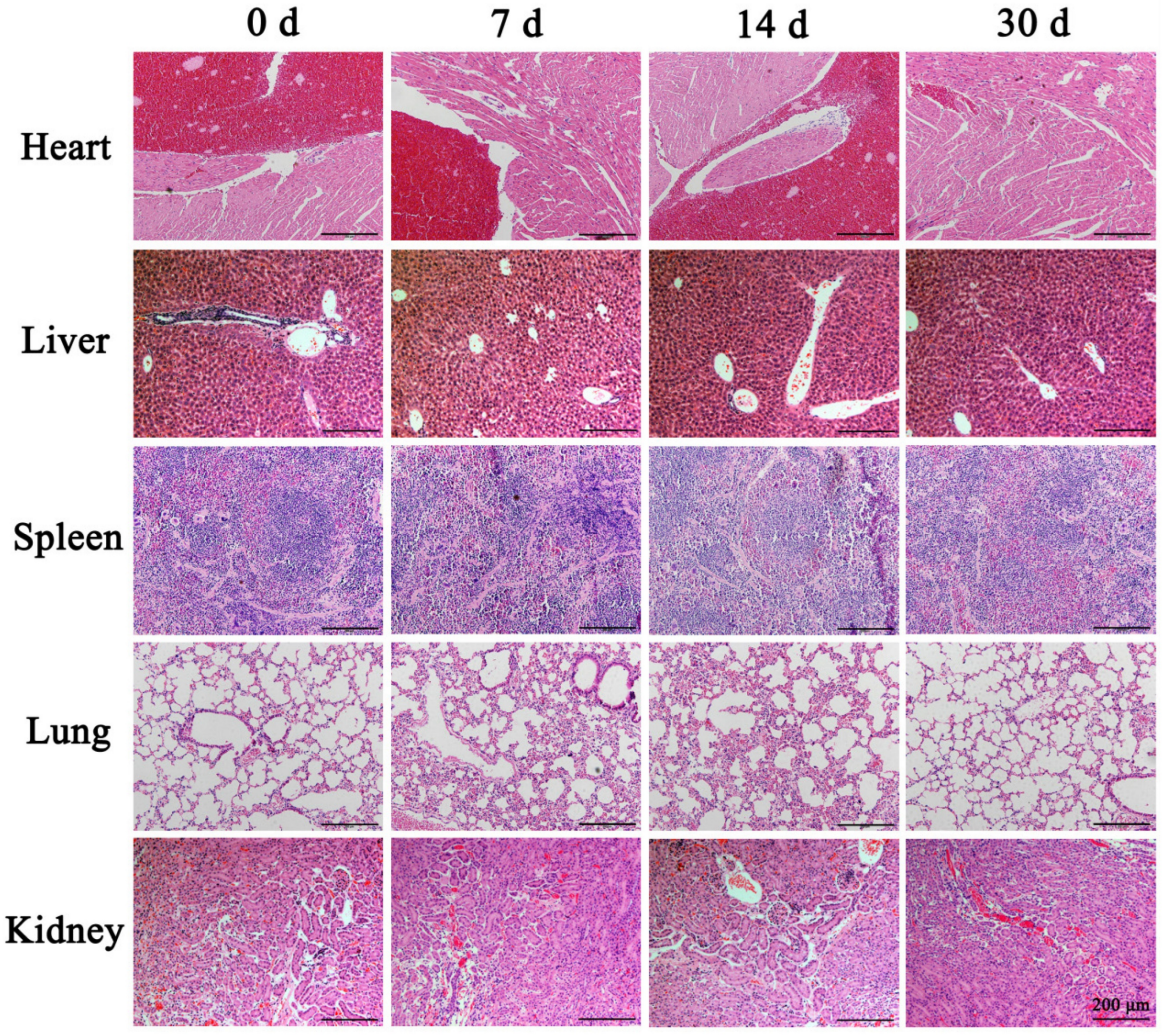
** Hematoxylin and eosin (H&E) staining of mouse tissues.** After the polyethyleneimine (PEI) nanohydrogel in PBS was injected into nude mice through the tail vein, the main organs (heart, liver, spleen, lung, and kidney) were stained with H&E at various time points to observe the nanohydrogel metabolism *in vivo*.

The results showed that the morphology of the various nude mice tissues did not change significantly at the different time points examined (Fig. 2). Therefore, the PEI nanohydrogel itself likely will not cause any harm to organs and tissues. The nanohydrogel prepared in this study can be metabolized and eliminated normally in nude mice and will not result in toxicity or side effects in normal tissues.

### UTMD parameters

**Figure 3:**
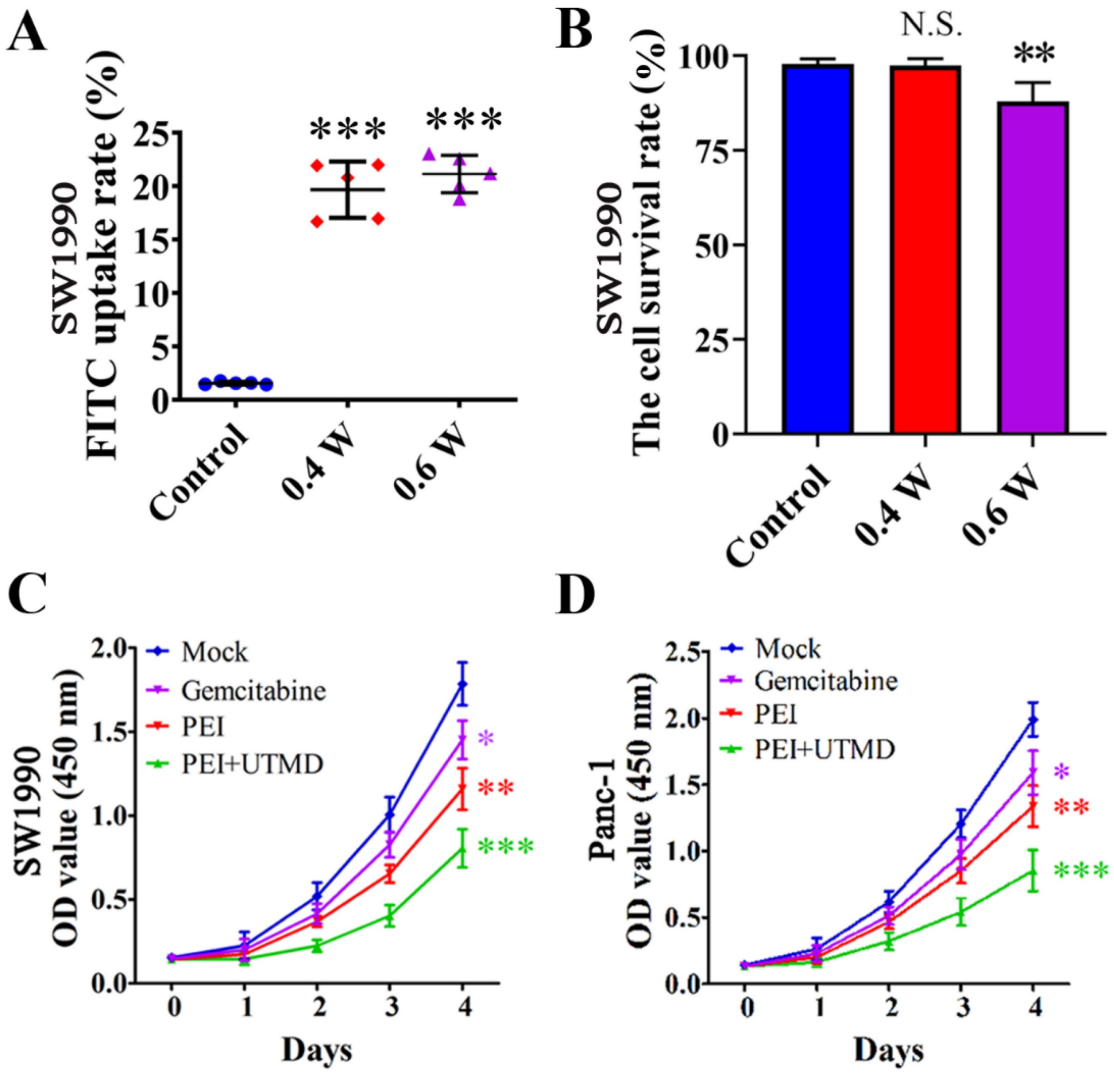
** Ultrasound targeted microbubble destruction (UTMD) parameter selection and its effects on pancreatic cancer cell proliferation. (A)** SW1990 treated with ultrasonic power of 0.4 W/cm^2^ and 0.6 W/cm^2^ showed higher FITC uptake rates compared with the control (*P*<0.001). **(B)** The cell survival rate of SW1900 with 0.6 W/cm^2^ group was significantly lower than that of control group (*P*<0.01), suggesting that 0.4 W/cm^2^ was the optimal UTMD parameter. **(C/D)** Polyethyleneimine (PEI) treatment significantly decreased the proliferation rates of SW1990 and Panc-1 cells, and adding UTMD could enhance that decrease further. Optical density (OD) values were measured at 450 nm by Cell Counting Kit-8 (CCK-8) assays at 0, 1, 2, 3, and 4 days. Each experiment was repeated three times, and the significance was analyzed by a student's t-test. Data are shown as the mean ± standard deviation (SD) (*P< 0.05, **P< 0.01 and ***P< 0.001 vs. Mock).

For the selection of UTMD parameters, it is generally believed that higher ultrasonic power will have a stronger effect on promoting cell uptake of drugs, but will cause greater damage to the cells. In contrast, lower ultrasonic power will have a weaker effect on drug uptake and result in less cell damage. Therefore, our goal was to maximize the balance between the FITC uptake rate and cell survival rates. The control group cells showed the lowest FITC uptake rate (1.55% ± 0.13%) and high cell viability (97.87% ± 1.31%). In contrast, the cells treated with ultrasonic power of 0.4 W/cm^2^ and 0.6 W/cm^2^ showed higher FITC uptake rates (19.66% ± 2.64% and 21.13% ± 1.75%, respectively) compared with the control cells (*P*<0.001) (Fig. 3A). The cell survival rate of the 0.6 W/cm^2^ group (87.99% ± 4.94%) was significantly lower than that of the 0.4 W/cm^2^ group (97.38% ± 1.92%) (*P*<0.01) (Fig. 3B). From these results, we chose 0.4 W/cm^2^ as the UTMD parameter for further *in vivo* and *in vitro* experiments.

### PEI nanohydrogel treatment can decrease PCC proliferation rates *in vitro*, which can be further decreased by adding UTMD

Cell proliferation rates were assessed by CCK-8 assays. As shown in Fig. 3C/D, these experiments revealed significantly reduced proliferation rates in both SW1990 and Panc-1 cells with PEI nanohydrogel treatment compared with control cells (*P*<0.01). Additionally, combining PEI treatment with UTMD could significantly reduce the proliferation rates even further (*P*<0.001).

### PEI nanohydrogel treatment can decrease PDAC tumor growth *in vivo*, which can be further decreased by adding UTMD

**Figure 4:**
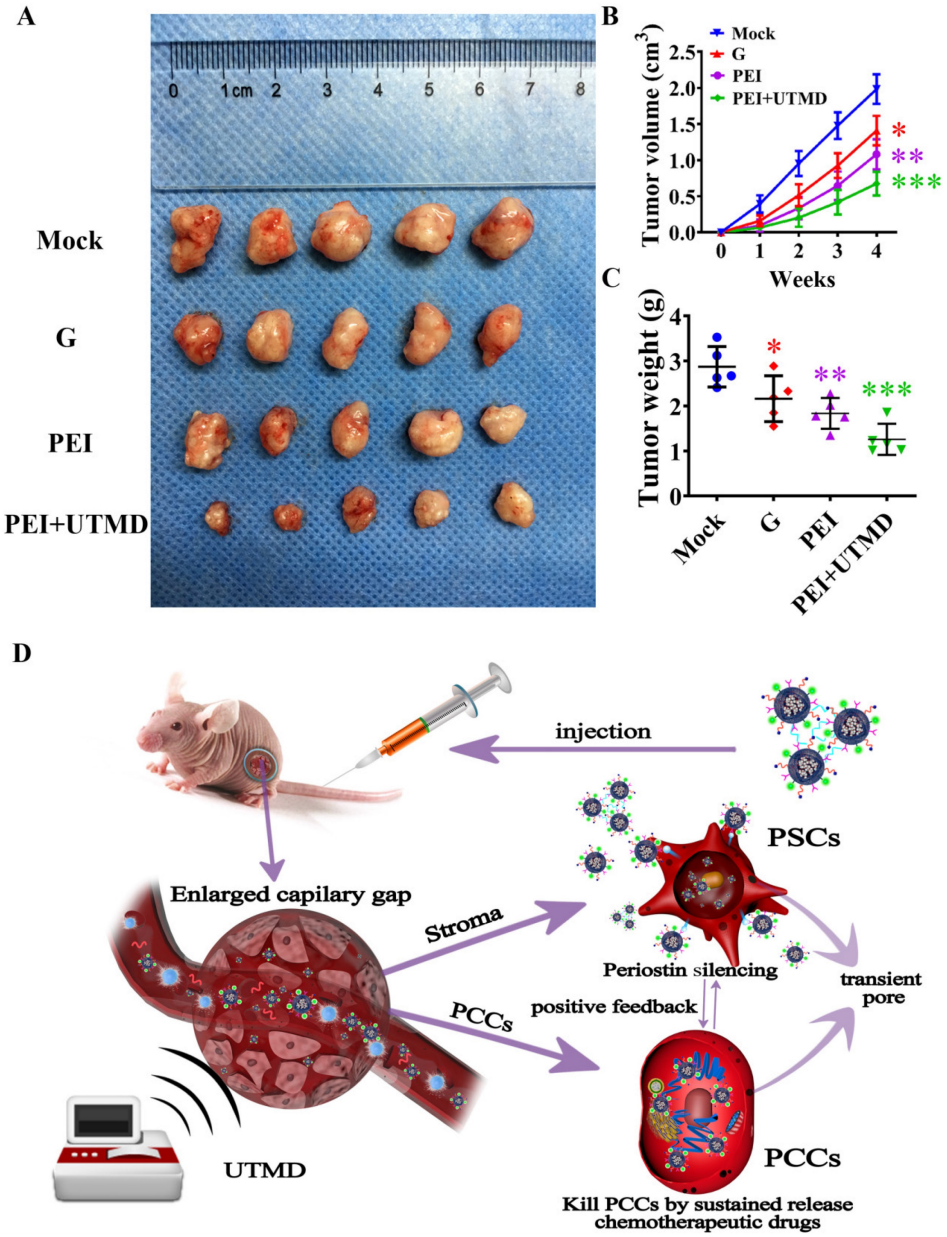
** Polyethyleneimine (PEI) nanohydrogel treatment decreases pancreatic ductal adenocarcinoma (PDAC) tumor growth, which can be further decreased by ultrasound targeted microbubble destruction (UTMD). (A)** 3×10^6^ SW1990 and 3×10^6^ PSCs were mixed in 20 μL PBS and subcutaneously implanted into the back of nude mice. One month later, the mice were sacrificed and all tumor xenografts were excised.** (B)** The PEI group showed decreased tumor volume compared with the gemcitabine alone and control groups (*P*<0.01). Additionally, the tumor volume of nude mice in the PEI + UTMD group was significantly smaller than that of the PEI group (*P*<0.001). **(C)** The tumor weight of the nude mice in the PEI group was significantly smaller than those of the gemcitabine alone and control groups (*P*<0.01). This lower tumor weight was decreased further by adding UTMD (*P*<0.001).** (D)** The schematic diagram of the action of PEI nanohydrogels in nude mice.

We then investigated the therapeutic effects of different forms of gemcitabine delivery routes *in vivo*. Firstly, the tumor size of each group after treatment was measured by two-dimensional ultrasound imaging. As shown in Fig. 4A, we could visually observe that the tumors from the nude mice treated with a combination of drug and gene silencing (PEI group) were smaller than those of the gemcitabine alone and control groups. The tumor volume was measured at different time points to evaluate the therapeutic effect of each group. As shown in Fig. 4B/C, the tumor volume and weight of the nude mice in the PEI group were also significantly smaller than those of the gemcitabine alone and control groups (*P*<0.01), indicating a better therapeutic effect of the combination therapy approach on pancreatic cancer. Moreover, compared with the PEI group, the tumor volume and tumor weight of nude mice in the PEI + UTMD group were smaller (*P*<0.001), indicating that adding UTMD could significantly increase the anti-tumor effects of PEI treatment. The action of PEI nanohydrogels in nude mice was illustrated in Fig. 4D.

### PEI nanohydrogel treatment increases PDAC tumor blood perfusion, which is further enhance by adding UTMD

**Figure 5:**
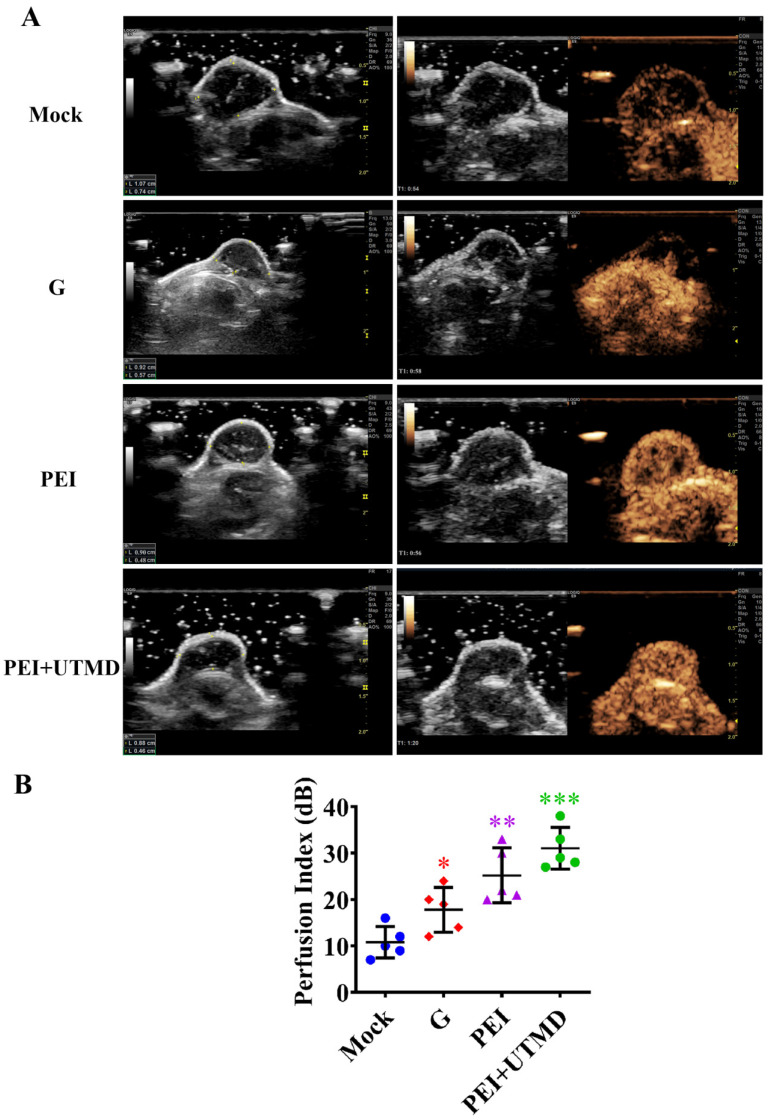
** Polyethyleneimine (PEI) nanohydrogel treatment increases pancreatic ductal adenocarcinoma (PDAC) tumor blood perfusion, which is further enhanced by adding ultrasound targeted microbubble destruction (UTMD). (A)** The blood perfusion of microvessels in tumors can be observed in real time using contrast-enhanced ultrasound (CEUS).** (B)** The quantitative analysis of blood perfusion in the different groups.

Examining blood perfusion in tumors after treatment is also an effective indicator for evaluating the therapeutic effect. This was observed in real time using CEUS. As shown in Fig. 5A, abundant intratumor blood perfusion was seen in the combined administration group, indicating a better prognosis. Further quantitative analysis of blood perfusion also verified these results (Fig.5B). Tumors in the PEI and PEI+UTMD groups showed more blood perfusion than those in the gemcitabine alone and control groups (*P*<0.01 and *P*<0.001, respectively). Moreover, the perfusion index of the PEI+UTMD group was significantly higher than that of the PEI group. This is mainly because of the direct contact between microbubbles and vascular endothelial cells in the circulatory system, which enhances vascular permeability through the acoustic pore effect and increases blood flow. This leads to a more effective uptake of the drug or gene product being delivered to achieve better therapeutic effects. These data suggest that UTMD can significantly enhance tumor suppression and improve the blood perfusion when treating pancreatic cancer.

### PEI nanohydrogel treatment combined with UTMD and MRI can support and improve the diagnosis and treatment visualization of pancreatic cancer *in vivo*

**Figure 6:**
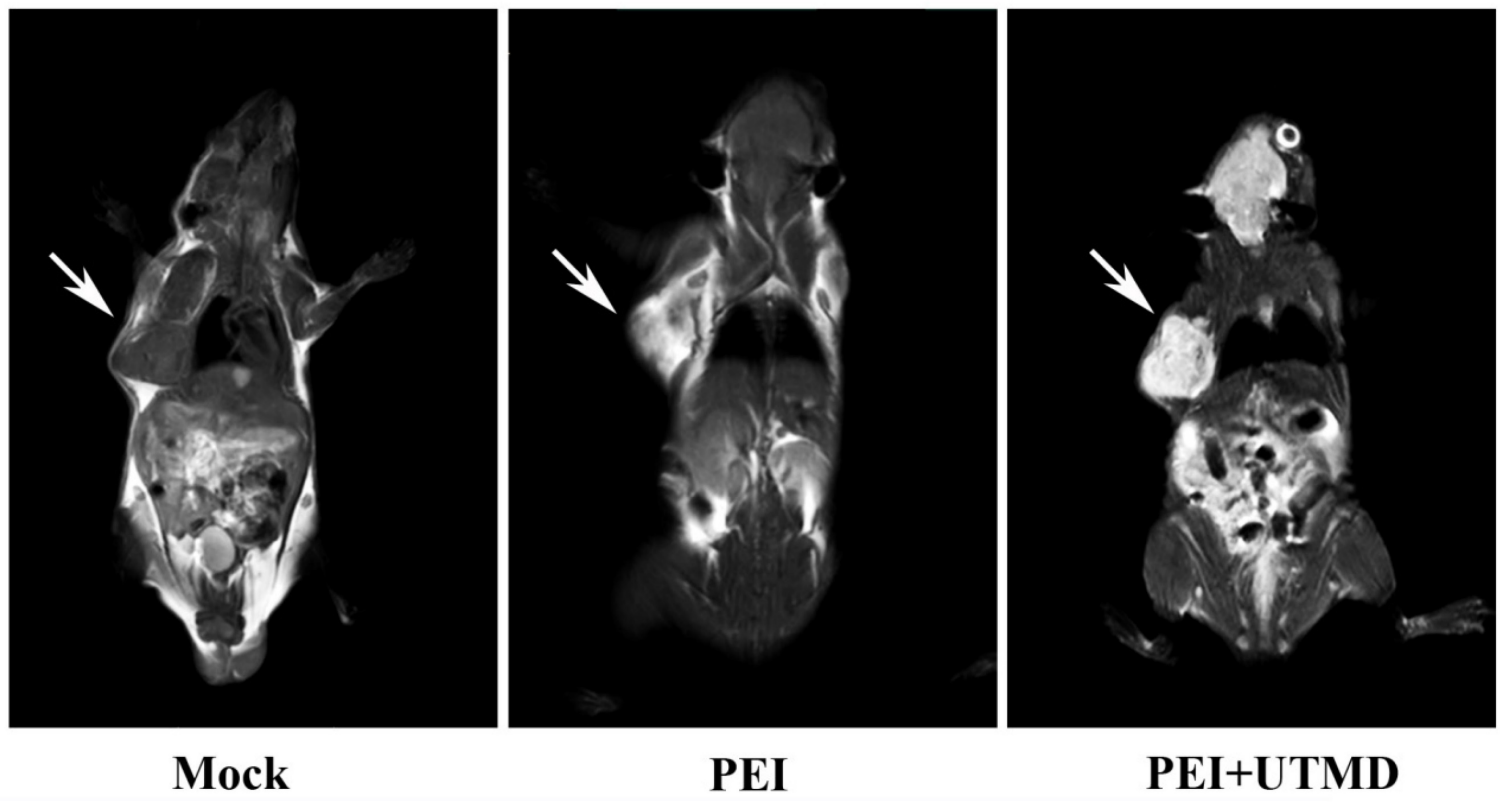
Polyethyleneimine (PEI) nanohydrogels have the potential for tumor magnetic resonance imaging (MRI).

MRI scans of the nude mice (Fig. 6) showed higher signal in the tumors following injection with PEI nanohydrogel materials compared with before injection. This finding indicates that our prepared PEI nanohydrogels have the potential for tumor MRI as contrast agents *in vivo*. This signal was enhanced when UTMD was added.

## Discussion

Pancreatic cancer is a highly malignant tumor with poor prognosis and high mortality^4^, ^45^. Because it is a hypovascular tumor, it is difficult for chemotherapy drugs to effectively enter and exert anti-tumor effects^46^, ^47^. Although nanohydrogels have been widely used for the delivery of anti-cancer drugs^48^, ^49^, there are few reports on the use of nanohydrogels as carriers for siRNA and drug delivery for treating pancreatic cancer. In this study, the results of our *in vivo* experiments suggested that adding UTMD could effectively increase the chemotherapeutic effect of the nanohydrogel carrier.

Most importantly, our experimental results demonstrated that there were differences in tumor vascular distribution and intratumoral blood perfusion between groups after treatment. Further quantitative analysis by CEUS showed that the blood perfusion of the tumor was greater in the samples with smaller tumor volume. In the control group, a substantial amount of necrosis was observed in the tumor because of the continuous tumor enlargement, resulting in little blood perfusion. This further indicated that pancreatic cancer tumors often have poor blood supply. Moreover, the CEUS data showed that the blood perfusion volume of the tumors treated with chemotherapy and periostin gene silencing combined with UTMD (PEI+UTMD) was significantly increased compared with the control group. The volume of these tumors was also significantly reduced, providing evidence for the enhanced efficacy when adding UTMD.

Perfusion was increased in the PEI+UTMD group, mainly because the microbubbles were in direct contact with vascular endothelial cells in the circulatory system and had increased vascular permeability from the acoustic pore effect. This led to a greater amount of drug/siRNA effectively entering the cells, resulting in a better therapeutic effect. Therefore, our data demonstrate that an increase in intratumoral blood perfusion allows chemotherapeutic drugs to enter cells and exert a stronger anti-tumor effect. These findings further suggest that UTMD can enhance the tumor suppressive effect of chemotherapeutic drugs and improve blood perfusion in tumors *in vivo*.

Additionally, this approach utilizes the multiple modification properties of nanohydrogels, including grafted Gd ions. This supports the use of tumor-targeted imaging with MRI and provides a new therapeutic visualization technology platform for pancreatic cancer chemotherapy.

## Conclusion

In this study, the local promotion function of UTMD technology was combined with drug and gene-loaded nanoparticles, which significantly improved the delivery efficiency of gemcitabine and periostin silencing, as well as the therapeutic effect against pancreatic cancer. Furthermore, the combination of gemcitabine and gene silencing can reduce the resistance of pancreatic cancer tumors to gemcitabine chemotherapy. Therefore, the co-delivery of gemcitabine and specific siRNAs using PEI can be significantly improved by adding UTMD, hence providing a promising strategy for pancreatic cancer clinical treatment.

## Abbreviations

PSCs: Pancreatic stellate cells
PEI: proposed polyethyleneimine
UTMD: ultrasound-targeted microbubble destruction
siRNA: small interfering RNA
MRI: magnetic resonance imaging
PDAC: Pancreatic ductal adenocarcinoma
PCCs: pancreatic cancer cells
FITC: fluorescein isothiocyanate
Gd: gadolinium
